# The general status of patients and limited physical activity as risk factors of Methicillin-resistant *Staphylococcus aureus* occurrence in long-term care facilities residents in Krakow, Poland

**DOI:** 10.1186/1471-2334-14-271

**Published:** 2014-05-18

**Authors:** Dorota Romaniszyn, Monika Pobiega, Jadwiga Wójkowska-Mach, Agnieszka Chmielarczyk, Barbara Gryglewska, Pawel Adamski, Piotr B Heczko, Dorota Ochońska, Malgorzata Bulanda

**Affiliations:** 1 Chair of Microbiology Jagiellonian University Medical College, Krakow, Poland; 2 Department of Internal Medicine and Gerontology, Jagiellonian University Medical College, Krakow, Poland; 3 Institute of Nature Conservation Polish Academy of Sciences, Krakow, Poland; 4 18 Czysta Street, 31-121 Krakow Poland

**Keywords:** Surveillance, Methicillin-resistant *Staphylococcus aureus*, Long-term care facilities, Physical activity limitations

## Abstract

**Background:**

The aim of this study was to investigate the epidemiology and resistance of methicillin-resistant *Staphylococcus aureus* (MRSA) isolates from long-term care facilities (LTCF) residents and to analyze the potential risk factors for MRSA occurrence, defined as MRSA colonization and/or infection.

**Methods:**

Point prevalence (PPS) and prospective incidence continuous study (CS) was carried out on a group of 193 residents in 2009-2010.

**Results:**

Overall MRSA occurred (with or without infection) among 17.6% of residents. There was 16 cases of infections with SA aetiology, of which 10 (58.8%) were caused by MRSA. The MRSA prevalence in PPS was 12.9%, in CS infection incidence rate was 5.2%. Factors associated with MRSA occurrence were: general status of patients, limited physical activity, wound infections (odds ratio, OR 4.6), ulcers in PPS (OR 2.1), diabetes (OR 1.6), urinary catheterization (OR 1.6) and stool incontinence (OR 1.2).

**Conclusions:**

Our data indicate a need for screening of MRSA before hospitalization or transfer to rehabilitation centres, especially in a group of residents with limitations in physical activity – i.e. with the highest risk of MRSA. Results also suggest the need for contact precautions in patients with high risk of MRSA occurrence, only. Focus on the high-risk population might be a solution for the cost-effective surveillance.

## Background

*Staphylococcus aureus* (SA) remains one of the most important potentially pathogenic microorganisms present in the human commensal flora, as it colonizes about 30%-40% of adults without any harm [1,2]. Prior colonization with SA is a risk factor for the development of an infection. A wide range of virulence factors and persistence of multidrug resistance can make the treatment of staphylococcal diseases challenging. An infection with methicillin-resistant strain results in greater length of hospital stay, higher mortality and increased costs [3]. Methicillin-resistant *Staphylococcus aureus* (MRSA) is no longer only a nosocomial pathogen. It has emerged as an important cause of community-associated infections. Over the recent years an increase in SA prevalence has been observed in many countries. A “new” reservoir of MRSA appeared: residents of long-term care facilities (LTCF). LTCF residents are a population at risk for MRSA because of age, age-associated morbidity, urinary devices and high rate of hospital contacts [4,5]. Repeated hospital admissions and transfer of patients with MRSA between hospitals are identified as causes of nosocomial MRSA acquisition [6]. Moreover, a majority of the residents rely on assistance for care and are bedridden, which is also a risk factor for MRSA carriage. An understanding of the prevalence and epidemiology of MRSA in LTCFs is essential for preparing guidelines for infection control. The epidemiology of SA remains unknown in Poland. Epidemiology and resistance of SA has not been studied among residents of Polish LTCFs.

The aim of this study was to investigate the prevalence and antibiotic resistance of SA isolates from Polish LTCF-residents, and to analyze potential risk factors for MRSA occurrence, defined as MRSA colonization and/or infection.

## Methods

The study consisted of two stages: the point prevalence and the prospective incidence study. Participation in the study was voluntary for both – LTCFs and residents.

### Point prevalence study

A 1-day point prevalence study (PPS) was carried out in October 2009 in 3 LTCFs in Krakow: 2 residential homes (RH) and 1 nursing home (NH). NH was defined as an institution where residents need 24 h/day medical or skilled nurses supervision and provide more intensive health care than RH, where residents are unable to live independently and require supervision or assistance with the activities of daily living. A resident was defined as a person who has stayed in LTCF for longer than 48 hours at the time of the study. Residents with mental disorders and residents younger than 65 years were not included in the study. The study protocol was approved by the Bioethical Committee of the Jagiellonian University (KBET/227/B/2012), conducted in accordance with the Declaration of Helsinki, and explained to the participants, who gave their written informed consent. Home-care staff completed a questionnaire about residents and risk factors that might be associated with MRSA/MSSA occurrence: presence of chronic diseases and other medical problems. Barthel Index (BI, a 10-item measure of disability based on daily activities and the score corresponds to the sum of all the points obtained, range 0-100 points) was obtained for all the residents participating in the study [7]. The Katz Index values were also obtained, which is an instrument to assess functional status and the ability to perform activities of daily living i.e. bathing, dressing, toileting, transferring, continence, feeding [8]. Physical dependence of the residents was classified according to a five-point scale (1-independent, 2- independent with falls, 3-limitations in movement, 4-bedridden, mobile, 5-bedridden, dependent). Data on the antibiotic and hospital exposure ≥7 days in the 3 months preceding the enrollment were collected. PPS study was performed to identify all residents with SA occurrence (cases of both colonization and infections). The aim of the PPS study was to collect demographic and clinical data about residents with MRSA (colonized and infected). Infections were defined according to McGeer’s criteria [9] and were detected by trained health personnel of LTCFs cooperating with the project worker. The kind of material collected for microbiological examination was dependent on the clinical status of the patients e.g. wound swabs, nasal swabs, sputum and others (data on all the studied residents). As a result, we wanted to assess how big a problem is MRSA in the studied LTCFs. An omission of the colonized residents without symptoms of the disease could have caused errors as to the real situation regarding the presence of the tested strain. Thus the aim was to collect demographic and clinical data of residents with MRSA (both sick and healthy carriers).

### The prospective incidence study

Continuous prospective infection control study (CS) was performed between December 1^st^, 2009 and November 30^th^, 2010 with standard McGeer definition protocol [9]. The study protocol was approved by the Bioethical Committee of the Jagiellonian University (KBET/227/B/2012), conducted in accordance with the Declaration of Helsinki, and explained to the participants, who gave their written informed consent. Infections were detected by trained health personnel of LTCFs cooperating with the project worker. The kind of material collected for microbiological examination was dependent on the clinical status of the patients e.g. wound swabs, pharyngeal swabs, sputum and others. Among 193 residents of LTCF participating in this research: 2 persons were excluded and 31 patients died. In the period between enrollment and each follow-up, data on potential factors that could increase the likelihood of MRSA acquisition were also collected. The study lasted 12 months. During that time period, the status of residents could have changed and new risk factors may have occurred. According to that, trained personnel of each LTCF observed patients on daily basis and noted all the changes in the condition of residents and information about hospitalizations – even those that were not related to the diagnosis or treatment of infections with MRSA aetiology.

### Statistical analysis

Relation between types of care, socio-demographic characteristics, probability and epidemiology of SA were analyzed with two main groups of statistical techniques. If the numerical parameters (age, length of stay *etc*.) were compared by the nominal character (type of care, form of infection *etc*.), ANOVA, which is the most powerful technique for dichotomic predictor was used. If the distribution of numerical characters did not fit the normal distribution, the most appropriate nonparametric alternative, which is the Wilcoxon test, was used instead. For the contingency of nominal characters frequency tests: chi-square (χ^2^) and likelihood ratio were used. The multivariate analysis of the influence of the risk factors on MRSA identification was conducted in two steps. First one was the logistic likelihood stepwise regression, backward model. In this step the risk factors for further most detailed analysis were chosen and then analyzed with General Linear Model with assumed binominal distribution of dependent variable and logit linked function. Due to the small number of infections in the PPS study – no statistical analysis was performed. P-values of <0.05 were considered significant. All analyses were performed using JMP®, Version 7. SAS Institute Inc., Cary, NC, 1989-2007.

### Bacterial isolates

Screening tests for SA were conducted at the beginning of the study (PPS). Swabs from anterior nares were collected from each enrolled resident and cultured for 24 h on blood agar plates. In PPS and CS, in case of an infection, various diagnostic specimens including tracheal/bronchial secretions and others were collected for culture and assessment of the microbial aetiology of infections (nasal swabs were not done continuously). Isolates were identified as SA by polymerase chain reaction (PCR) [10].

### Antimicrobial susceptibility

All isolates were tested using disk diffusion antimicrobial susceptibility methods on Mueller-Hinton agar plates according to the current guidelines of the European Committee on Antimicrobial Susceptibility Testing (http://www.eucast.org/clinical_breakpoints/). Antibiotics used in this study included erythromycin (2 μg), clindamycin (15 μg), moxifloxacin (5 μg), doxycycline (30 μg), norfloxacin (10 μg), tobramycin (10 μg), gentamycin (10 μg), amikacin (30 μg), ciprofloxacin (5 μg), mupirocin (200 μg). All disks were obtained from Oxoid (Basingstoke, United Kingdom). Etest for vancomycin (bioMerieux, France) was also performed for all the isolates.

The macrolide-lincosamide resistance phenotype of the isolates was determined according to previously published protocol [11].

### DNA isolation and PCR-based detection of genes

The total DNA was isolated from bacterial strains with Genomic Mini (A&A Biotechnology, Poland) according to the manufacturer’s recommendations. Multiplex PCR amplification was used to specify the strain and to detect the presence of *mec*A gene using previously published primers [10]. As controls, *S. aureus* ATCC 33591 (*mec*A+) and *S. aureus* ATCC 25923 (*mec*A−) were employed. PCR amplification of a 456 bp fragment of the *mup*A gene was performed [12]. Relevant positive and negative controls were included. The PCR equipment used was a DNA Engine Peltier Thermal Cycler (BioRad). Bands were visualized using UVP GelDocIT Imaging System after 1.5%-TAE-agarose electrophoresis (70 min, 90 mV) with ethidium bromide (BioRad). GeneRuler DNA-ladder 1 kb (Fermentas) was used as a size marker.

### Pulsed-field gel electrophoresis (PFGE)

The process of conducting the analysis of genetic similarity of SA isolates was performed in accordance with the previously published protocol using CHEF-DR III apparatus (Bio-Rad). Profiles were analyzed with Molecular Analyst Fingerprinting Software (BioRad) [13]. Dendrograms were generated using band-based Dice similarity coefficients and analysed with the application of the criteria by Tenover *et al*[14].

## Results

### Characteristics of population

In total 3 LTCFs from Krakow agreed to participate in this study: 2 residential homes and 1 nursing home. These facilities served 520 residents of which 193 were included to the study. Included were only residents with appropriate age, who agreed to participate in the study. Residents younger than 65 years or persons with psychiatric disorders or those who did not give consent were excluded. From the RHs about 90% of residents were included, differently from NH, where only 25% residents were included (about 20% of residents fulfilling the criteria of the study refused to participate in the study). Of the residents, 86 (39.6%) stayed at residential homes (RH), while 107 stayed at the nursing home (NH). Studied sample corresponded to 2.6% of total LTCF population in Malopolska in 2010 [15].

The average age for the population of whom 63.2% were female was 76.2 years (SD ± 10.5, 95% confidence interval, CI. 72.3-77.9). The median length of LTCF stay was 3 years (RHs: 4; NHs: 3), range: from 2 months to 26 years.

#### MRSA infections and colonization

There were 16 cases of infections of SA aetiology, 10 (62.5%) of which were caused by MRSA (8 wound infections and 2 pneumonia). In the PPS study, there were 4 cases of MRSA infections: in 2 residents with isolates both from wounds and the nasal swabs; and in 2 residents only from wounds. When it comes to the 6 MRSA infections described in CS: 1 resident with two infections (pneumonias) of different MRSA aetiology was observed. In CS the MRSA incidence rate was 5.2% and incidence density rate was 0.2/1000 residentdays.

Nasal swabs from 193 residents were obtained. Overall in PPS, 56 residents (29.0%) were colonized with SA, 23 of the isolates (41.1%) were methicillin-resistant. The prevalence of MRSA colonization was highest in NH (16.8%), whereas in RH1 and RH2 the prevalence was much lower (6.7% and 3.6% respectively). The MRSA prevalence in PPS was 12.9%.

### Risk factor analysis

In the PPS the MRSA strains were found in 4 cases of wound infections. Nasal colonization was statistically significant and associated with the type of care (NH vs. RHs), the presence of bladder catheter, urinary incontinence, dysphagia or gastric feeding tube, age, length of stay, Barthel scale and limited mobility. There were no difference between RH and NH residents and persons with urinary catheters, and others (Table 1).

**Table 1:** Characteristics of residents with nasal colonization of MRSA in the PPS (n = 193)

**Characteristics of the study group**	**No of participants**		**% MRSA**	**Univariate analysis**		**Multivariate analysis**	
	**With MRSA**	**Without MRSA**		**OR; 95% CI**	**p-value**	**OR; 95% CI**	**p-value**
Gender				0.38; 0.13-1.07	-	1.83; 0.623-6.226	0.0405
Male	5	72	6.9				
Female	18	98	25.0				
Type of care				0.31; 0.11-0.86	0.0011	2.76; 0.102-9.787	-
RHs	5	81	6.9				
NH	18	89	25.0				
Diabetes				2.55; 1.049-6.18	-	4.84; 0.786-25.933	-
Yes	11	45	15.3				
No	12	125	16.7				
Urinary incontinence: diapers				1.49; 0.61-3.59	0.0196	1.83; 0.565-11.487	-
Yes	10	58	13.9				
No	13	112	18.1				
Urinary permanent catheterization				2.3; 0.89-5.89	0.0014	1.267; 0.231-7.991	-
Yes	8	32	11.1				
No	15	138	20.8				
Dysphagia				0.79; 0.22-2.86	0.0118	0.68; 0.103-53.815	-
Yes	3	27	4.2				
No	20	143	27.8				
Nasogastric tube				55.63; 6.49-477.09	0.0228	1.93 e-7; 0.0-2.64 e-31	-
Yes	7	19	9.7				
No	16	151	22.2				
Ulcers				4.1; 0.95-17.68	0.0482	0.21; 0.039-1.273	-
Yes	3	6	4.2				
No	20	164	27.8				
Feeding via gastrostomy tube				3.94; 1.42-10.92	0.041	1.91; 0.123-8.134	-
Yes	7	17	9.7				
No	16	153	22.2				
Age mean ± SD (95% CI) [years]	79.9 ± 116 (75.3-84.5)			NA	-	NA	0.0132
Length of stay mean ± SD (95% CI) [years]	3.8 ± 1.2 (1.4; 6.1)			NA	0.0151	NA	-
Barthel’s Index, mean ± SD (95% CI)	21.3 ± 25.5 (11.2-31.4)			NA	<0.001	NA	-
Katz scale, mean ± SD (95% CI)	1.6 ± 1.9 (0.9-2.4)			NA	0.004	NA	-
Physical activity* mean ± SD (95% CI) [scale 1-5]	3.7 ± 1.3 (3.2-4.2)			NA	<0.0013	NA	0.0009

*Physical dependence of the residents was classified according to a five-point scale (1-independent, 2- independent with falls, 3-limitations in movement, 4-bedridden, mobile, 5-bedridden, dependent).

SD – standard deviation; 95% CI – 95% confidence interval; NA not applicable.

The results from the analysis for risk factors associated with MRSA nasal colonisation in the PPS are presented in Table 1. Majority of risk factors investigated (such as: sex, age, hospitalization before our study, obesity, stroke and skin changes) were not significantly associated with SA or MRSA occurrence. Factors from the single variable analysis that exhibited association with MRSA colonisation were the general status of patients, expressed with Barthel and Katz Indexes, limited physical activity, ulcers in PPS, urinary catheterization. Patients with MRSA were not hospitalized more often than patients without MRSA. Staying in the RHs, compared to the NH, significantly reduced the risk of MRSA. Age of residents was not linearly correlated with MRSA. Similar relationship was observed in the analysis of the impact of length of stay on the risk of MRSA: the risk was significant and more frequently observed in people who stayed for not longer than 3 years (OR 162, 95%CI 38.89-674.84, p = 0.010).

MRSA was observed in 25 of 38 residents with physical activity limitations. Prevalence of MRSA in this group was 65.8% (relative risk, RR 12.1). Independent residents without limitations had significantly lower MRSA rate.

In CS MRSA was sought only in residents with symptoms of infection. It was isolated significantly more often among residents previously colonized, staying in NH, with leg ulcers, low Barthel score and limited mobility.

### Multivariate analysis

During logistic likelihood (log likelihood, R^2^_whole model_ = 0.1995) stepwise regression 6 factors for future analysis were selected: infections in continuous study, gender, age, wounds in PPS, physical activity and ulcers. Significant for the nasal colonisation of MRSA were: gender, age and physical activity (Table 1). The independent predictors of MRSA occurrence were: age, physical activity and ulcers – without effect of gender (Table 2).

**Table 2:** Independent predictors of MRSA occurrence

**Characteristics of the study group**	**OR**	**95% CI**	**p-value**
Gender	2.21787	0.77587-7.35603	-
Type of care	2.820409;	0.85361-10.49075	-
Diabetes	2.49199;	0.97879-6.32626	-
Urinary incontinence: diapers	0.368843;	0.087983-1.317818	-
Urinary permanent catheterization	2.711179;	0.75883-11.365892	-
Dysphagia	2.630631;	0.20216-23.15387	-
Nasogastric tube	7.32428;	0.10412-9.604325	-
Ulcers	0.084993;	0.014193-0.488536	0.0051
Feeding via gastrostomy tube	1.496 e-7;	0.000-0.45512	-
Age mean ± SD (95% CI) [years]	NA		0.0132
Length of stay	NA		-
Barthel’s index	NA		-
Katz scale	NA		-
Physical activity*	NA		0.0009

*Physical dependence of the residents was classified according to a five-point scale (1-independent, 2- independent with falls, 3-limitations in movement, 4-bedridden, mobile, 5-bedridden, dependent).

SD – standard deviation; 95% CI – 95% confidence interval; NA not applicable.

### Bacterial strains and resistance

SA isolates from infections were resistant mainly to fluoroquinolones, tobramycin and amikacin. Among isolates from nasal swabs (colonization), 7 were clindamycin and erythromycin resistant (constitutive mechanism of macrolide-lincosamide resistance was detected in 9 MRSA and 4 SA isolates). Five isolates with M phenotype (resistant to erythromycin but susceptible to clindamycin) were found*.* Of the isolates tested, 7 exhibited intermediate resistance to mupirocin. Only one of these isolates showed the presence of *mup* gene. The results of susceptibility testing are shown in Table 3. There was no isolates resistant to vancomycin.

**Table 3:** Antimicrobial resistance of SA isolates in the infections and in colonization

**Antimicrobial**	**Wound infections [n = 14] no/%**		**Other infections [n = 2] no/%**		**Nasal swabs [n = 56] no/%**	
Erythromycin	6	42.9	1	50.0	12	21.4
Clindamycin	5	35.7	1	50.0	7	12.5
Norfloxacin	11	78.6	2	100.0	30	53.6
Ciprofloxacin	11	78.6	2	100.0	30	53.6
Gentamicin	4	28.6	1	50.0	7	12.5
Tobramycin	9	64.3	2	100.0	28	50.0
Amikacin	9	64.3	2	100.0	28	50.0
Mupirocin	0	0.0	0	0	0	0.0
Moxifloxacin	11	78.6	2	100.0	26	46.4
Methycillin*	8	57.1	2	100.0	23	41.1
Vancomycin**	0	0.0	0	0.0	0	0.0

*Resistance to methycillin was measured with PCR and therefore methycillin resistance means here *mecA* carriers.

**vancomycin resistance was measured with e-test.

**Figure 1:**
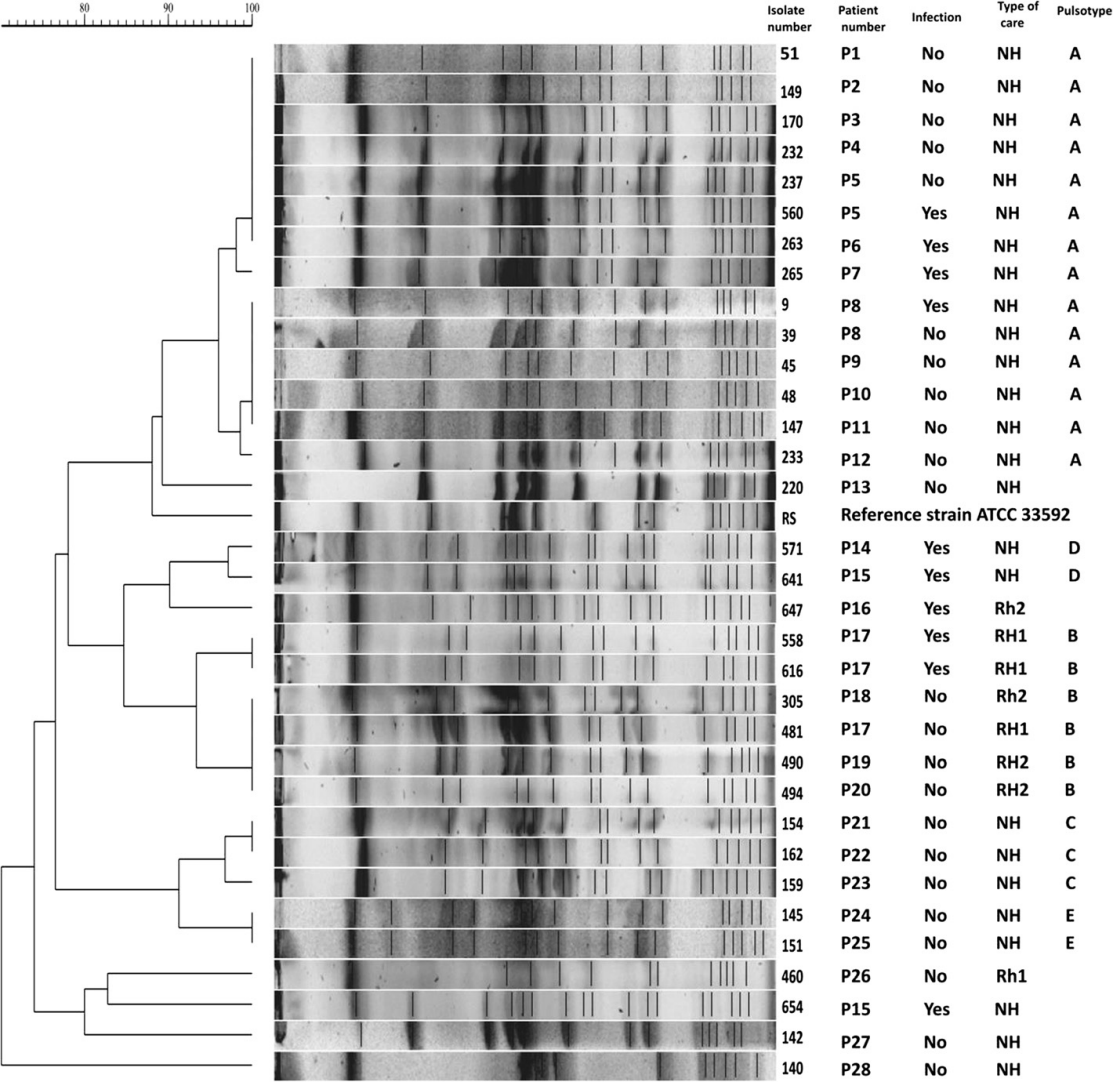
**Pulsed-field gel electrophoresis (PFGE) dendrogram of methycillin-resistant Staphylococcus aureus isolates from the long-term care facilities residents (NH – Nursing Home, RH – Residential Home).** PFGE clusters with >92% similarity are indicated.

Analysis of *Sma*I macrorestriction profiles of the 33 MRSA isolates revealed 13 different PFGE patterns (Figure 1). Results of MRSA-PFGE showed that 6 residents had a unique strain. Five PFGE patterns that differed by no more than three bands were identified. Closely related strains differing in their patterns by three bands or less were clustered in 5 genomic groups. In each group, strains had more than 92% relatedness. Pattern A was recovered in 14 strains, pattern B in 6 strains, pattern C in 3 strains, pattern D in 2 strains, pattern E in 2 strains. The patterns A, D, E were exclusively identified in strains isolated from NH residents, pattern B was mainly identified in strains from RH1 and RH2. Strains belonging to group A were recovered in one unit, showing the spread of clones in the LTCF.

Analysis of *Sma*I macrorestriction profiles of the MSSA isolates revealed that there was no similarity between patterns.

## Discussion

This is the first Polish study of infections and colonization of MRSA among LTCFs residents in Krakow, showing results from different included populations of residents, some of them being more, others at a lower risk of infection.

In general, LTCF residents represent a population at risk for MRSA [4]. Understanding the epidemiology of community and healthcare associated MRSA is essential for guiding new control programs. The prevalence of MRSA reported in our study is higher than rates which have been recorded in other European surveys. For example, in Germany the prevalence of MRSA was 7.6% [4]. Significantly lower rates (1.1%-2.3%) have been reported earlier in Germany [5]. Higher rates (about 20%) have been reported in the United Kingdom and in Northern Italy [16,17]. Studies conducted in Spain revealed, that about 47% of the MRSA carriers had persistent colonization for six or more months and the annual incidence of MRSA acquisition in this study was reported to be about 16.8% [18]. In Swedish nursing homes about 50% of residents were colonized with SA, but none of the isolates were methicillin-resistant [19]. The prevalence of MRSA colonization in Dutch nursing homes has been also evaluated and was very low (0.33%, 95% CI 0.14-0.74) [20]. The Netherlands have one of the lowest levels of MRSA in Europe, attributed to a strict policy on antibiotic usage and guidelines for MRSA control in hospitals [21].

On the other hand, only 26.3% of studied Polish residents were colonized with SA, which reflects the SA colonization rate in the population.

In Californian nursing homes about 95% of MRSA isolates were resistant to erythromycin and 74% to clindamycin [22]. In our study the proportions were much lower (42.4% and 27.3%, respectively). Similar numbers of MRSA isolates were resistant to gentamycin in both studies (11% in Californian nursing homes and 12.1% in Polish LTCF) [22].

An outbreak which was observed in the UK and which was associated with transmission of mupR-MRSA showed that the hospital may have been exporting mupR-MRSA into the community [23]. A lot of other studies also suggested that resistance to mupirocin is increasing. Fortunately, none of the isolates in this study were resistant to mupirocin.

Although in our study there was no strong correlation between age and MRSA occurrence, a special trend was observed: residents aged >90 years were more likely to show MRSA occurrence. In Gloucestershire residents aged <81 years were significantly more likely to carry MRSA, while MSSA carriage was significantly higher in residents aged >90 years [24].

The case of recent hospitalization in an acute ward was not significantly associated with MRSA carriage in French LTCF [25], as in our study. Other studies, however, have identified hospitalization as a risk factor for MRSA colonization [5,26]. Being bedridden and dependent were found to be risk factors for MRSA carriage. This finding suggests that residents who rely on assistance for care were at a higher risk of MRSA carriage, and it may be connected with numerous procedures which need to be done for the resident: washing and other activities related to personal hygiene, change of linen and clothing, feeding, maintaining the process of changing positions or moving. On the other hand, bedridden residents are also exposed to wounds, such as pressure sores and other skin changes due to lack of mobility (lying), skin condition (thin, dry, inflexible, requiring intensive care) and the permanent presence of dressings and/or diaper. Residents with the Barthel Index that equalled 0 were accommodated in single apartments, while other residents, even with limitations – in 2, 3 or 4-bedroom apartments. In such rooms, bedridden residents were accommodated together with independent residents. Length of LTCF stay was 2-times shorter for residents with MRSA, which is not surprising, because such residents had the lowest Barthel scores or limited mobility, that increase the risk of infection [27] and mortality. This was indicated in the single variable analysis, as low values of Barthel and Katz Indexes. Other usual risk factors for MRSA carriage, such as invasive procedures and skin lesions/wounds, were associated with carriage, as it was for French or German LTCFs [4,25].

These results, combined with PFGE data, may suggest that residents are acquiring MRSA by cross-infection via the staff members and demonstrates the need for more strict hygiene standards in care homes. On the other hand, horizontal transmission was the predominant route of transmission of microorganisms in the analyzed units.

The prevalence of MRSA in nasal colonization was highest in NH which may be caused by worse general status of residents – most of them were bedridden, with low Katz and Barthel Index, which means that they need a lot of assistance in every-day life. The PFGE patterns of NH MRSA suggests that there was one dominating clone which colonized most of the residents (8 of 18 nasal isolates had the same pattern): all these residents were characterized by low Katz-scale (less than 2) and Barthel Index (less than 40). It is worth noting, that the lack of significance of commonly used indexes in univariable analysis (Barthel, Katz) may be an effect of hidden correlation between those indexes and other studied risk factors. Influence of age, however significant, was rather a low predictable. There is a possibility to conclude that residents with physical activity level 1 and 2 are less predisposed to MRSA isolation than residents with activity levels 4 and 5.

Unfortunately in all the studies cited above, the risk of MRSA occurrence (or colonization) associated with the general status. Possibility of using such common type of indicators (limitations of physical activity) should be considered in epidemiological studies, such as the epidemiology of infection and the occurrence of multi-drug resistant organisms.

Our data indicate a need for MRSA screening, e.g. prior to admission to the LTCF, hospital or to the rehabilitation centre, especially in group of residents with physical activity limitations – i.e. with the highest risk of MRSA occurrence. Such targeted surveillance can be particularly important in countries with limited resources in infection control, such as Poland. Focus on the high-risk population might be a solution for the cost-effective surveillance.

In Poland, there are no recommendations for the prevention of MRSA transmission in LTCFs. The policy of most facilities is to admit MRSA carriers (with or without infections), whereas our data indicate the need for contact precautions in patients with high risk of MRSA. It is especially important in case of necessity to hospitalize or to transfer to rehabilitation centres. It can be narrowed down to the selected groups of residents with physical activity limitations – with the highest risk of MRSA.

Several potential limitations should be considered in the interpretation of data presented here. First, the number of patients included was low (only 193 residents from 3 LTCFs). It was due to mistrust of the residents and the negative attitude of the personnel. For these reasons the number of isolates was also low. This may be due to the characteristics of the Polish population staying in LTCFs presented in this research. A second limitation was that the studied population did not include all the residents of selected LTCFs, but was limited to those residents who gave written consent. Thus the research was focused on generally healthier residents of LTCFs, which could have influenced the observed data. The results would probably be worse if the survey covered the entire population of the LTCF residents. This observation, however, also indicates that the MRSA occurrence risk among the ‘healthier’ group of residents is significant, and all possible methods for reducing the prevalence and improving the prognosis in this group of older people should be recommended.

However, this is the first Polish surveillance conducted in LTCFs, and further research should be done.

## Conclusions

Our data indicate a need for screening of MRSA before hospitalization or transfer to rehabilitation centres, especially in a group of residents with limitations in physical activity (bedridden, mobile or depend) – i.e. with the highest risk of MRSA. Results also suggest the need for contact precautions in patients with high risk of MRSA occurrence, only. Focus on the high-risk population (bedridden, mobile or depend) might be a solution for the cost-effective surveillance.

## Abbreviations

BI: Barthel Index; CI: Confidence interval; CS: Continuous study; DNA: Deoxyribonucleic acid; LTCF: Long-term care facilities; MRSA: Methicillin-resistant *Staphylococcus aureus*; NH: Nursing home; OR: Odds ratio; PCR: Polymerase chain reaction; PFGE: Pulsed-field gel electrophoresis; PPS: Point prevalence; RH: Residential home; RR: Relative risk; SA: *Staphylococcus aureus.*

## Competing interest

The authors declare that they have no competing interests.

## Authors’ contributions

DR carried out the antimicrobial susceptibility studies and drafted the manuscript. MP carried out the molecular genetic studies (PFGE) and drafted the manuscript. JWM designed the study, analyzed and interpreted the data, performed the statistical analysis and financially supported the study. ACh carried out the molecular genetic studies (DNA isolation and PCR-based detection of gene) and drafted the manuscript. BG conceived of the study, and participated in its design and coordination and helped to draft the manuscript. PA performed the statistical analysis. DO carried out the antimicrobial susceptibility studies. PBH conceived of the study, and participated in its design and helped to draft the manuscript. MB helped to draft the manuscript. All authors read and approved the final manuscript.

## Pre-publication history

The pre-publication history for this paper can be accessed here:

http://www.biomedcentral.com/1471-2334/14/271/prepub
